# Preferential amplification of a human mitochondrial DNA deletion *in vitro* and *in vivo*

**DOI:** 10.1038/s41598-018-20064-2

**Published:** 2018-01-29

**Authors:** Oliver M. Russell, Isabelle Fruh, Pavandeep K. Rai, David Marcellin, Thierry Doll, Amy Reeve, Mitchel Germain, Julie Bastien, Karolina A. Rygiel, Raffaele Cerino, Andreas W. Sailer, Majlinda Lako, Robert W. Taylor, Matthias Mueller, Robert N. Lightowlers, Doug M. Turnbull, Stephen B. Helliwell

**Affiliations:** 1 Wellcome Centre for Mitochondrial Research, Institutes of Neuroscience and Cellular and Molecular Bioscience, Newcastle University Medical School, Newcastle University, Newcastle upon Tyne, NE2 4HH Tyne and Wear UK; 2 Novartis Institutes for BioMedical Research, Novartis Campus, Basel, CH-4056 Switzerland; 3 Institute of Genetic Medicine, Newcastle University, Newcastle, United Kingdom

## Abstract

We generated induced pluripotent stem cells (iPSCs) from patient fibroblasts to yield cell lines containing varying degrees of heteroplasmy for a m.13514 A > G mtDNA point mutation (2 lines) and for a ~6 kb single, large scale mtDNA deletion (3 lines). Long term culture of the iPSCs containing a single, large-scale mtDNA deletion showed consistent increase in mtDNA deletion levels with time. Higher levels of mtDNA heteroplasmy correlated with increased respiratory deficiency. To determine what changes occurred in deletion level during differentiation, teratomas comprising all three embryonic germ layers were generated from low (20%) and intermediate heteroplasmy (55%) mtDNA deletion clones. Regardless of whether iPSCs harbouring low or intermediate mtDNA heteroplasmy were used, the final levels of heteroplasmy in all teratoma germ layers increased to a similar high level (>60%). Thus, during human stem cell division, cells not only tolerate high mtDNA deletion loads but seem to preferentially replicate deleted mtDNA genomes. This has implications for the involvement of mtDNA deletions in both disease and ageing.

## Introduction

Understanding the pathogenetic mechanisms involved in the formation and subsequent clonal expansion of mitochondrial DNA (mtDNA) mutations in human disease is challenging. Generation of animal models with mtDNA mutations is difficult because of the inability to manipulate the mitochondrial genome^1^ and whilst studies in human tissues can give valuable insights, the inability to study clonal expansion at different stages of human development is an important limitation. For this reason there has been considerable interest in developing induced pluripotent stem cell (iPSC) lines to study mtDNA disease. The majority of these studies have focussed on point mutations of the mitochondrial genome and have given important insights into pathogenetic mechanisms and for the development of potential therapies^2–10^.

The commonest sporadic defect of mtDNA seen in patients are single large-scale deletions, accounting for approximately 17% of all adult patients with mitochondrial disease^11^. The phenotypes due to single, large-scale mtDNA deletions range from the often fatal Pearson syndrome in infancy, Kearns Sayre syndrome in childhood and adolescence, to late onset chronic progressive external ophthalmoplegia^12^. The severity of the phenotype relates directly to the level of deleted mtDNA and size of the deletion^13^. There are no good animal models of single, large-scale mtDNA deletions and very limited iPSC studies enabling us to explore how a sporadic mtDNA mutation can accumulate from a deletion in a single molecule of mtDNA to the high mutation loads and diverse tissue involvement seen in human disease.

To increase our understanding of single, large-scale mtDNA deletion disease and clonal expansion we generated iPSC lines with different levels of heteroplasmy of ~6 kb mtDNA deletion, as well as two cell lines containing a mtDNA point mutation with different levels of heteroplasmy. The cell lines with deleted mtDNA consistently showed an increase in mutated mtDNA during cell culture in contrast to those with a point mutation in which the level of heteroplasmy remained stable. In addition, when we generated teratomas from the deleted cell line we showed high levels of heteroplasmy significantly greater than the original cell line, a phenomenon not seen previously with mtDNA point mutations^4^. This clonal expansion of deleted mtDNA may have important implications for our understanding of deletion mtDNA disease and may provide a useful model for future studies.

## Results

### Heteroplasmy segregates during reprogramming

**Figure 1:**
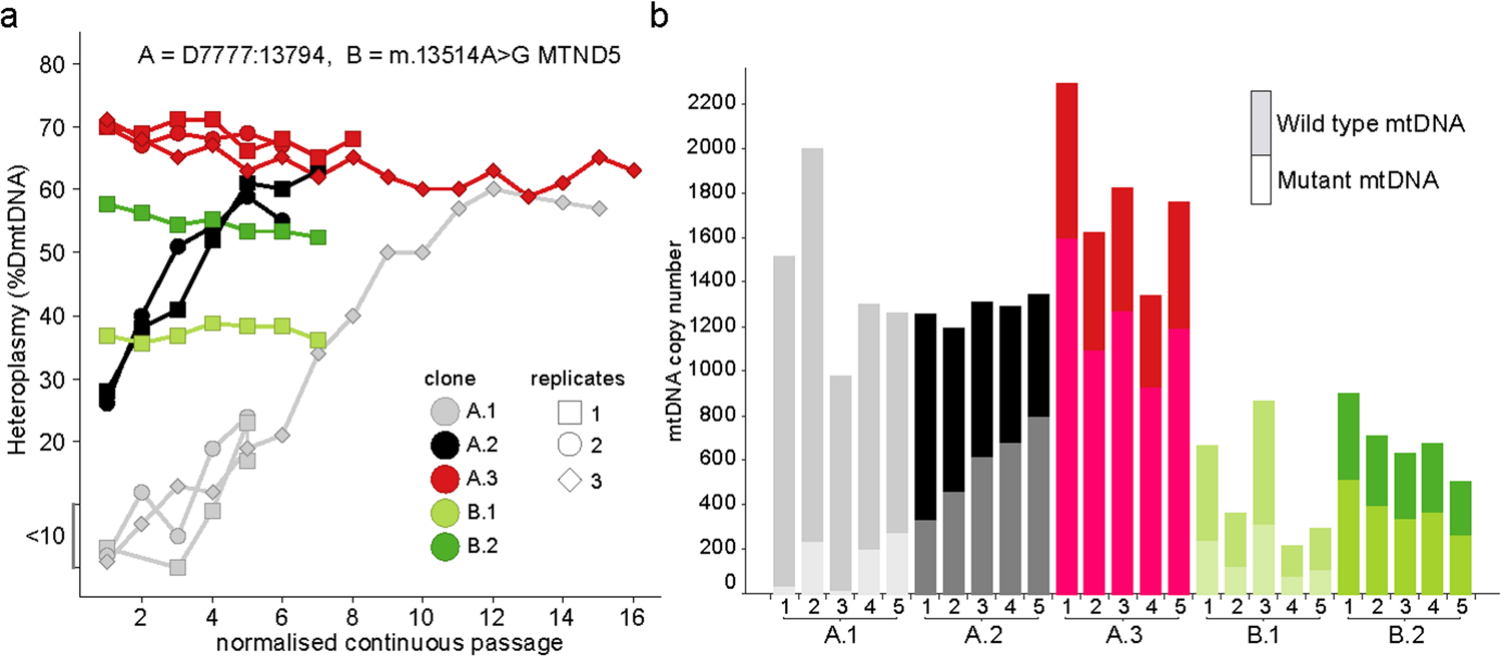
Molecular characterisation of mtDNA heteroplasmy and copy number after iPSC generation. (**a**) Heteroplasmy during continuous passages of iPSCs, following reprogramming of two patient fibroblasts carrying mtDNA Δ7777:13794 at 17% (A), and m.13514 A > G at 55% (B). A.1 (grey), A.2 (black), A.3 (red), B.1 (light green) and B.2 dark green) clones were continuously passaged and heteroplasmy assessed. (**b**) Values for wild type and mutant copy number (CN) determined from iPSCs from each replicate of the first 5 continuous passages in (**a**).

We reprogrammed two mitochondrial patient fibroblast cell lines to iPSCs using Sendai vectors expressing c-Myc, Sox2, Klf4 and Oct4^14^. Patient A was a female child with Pearson’s syndrome caused by a ~6.0 kb single, large-scale mtDNA deletion; Patient B was a female child with Complex-I deficient Leigh syndrome and a heteroplasmic m.13514 A > G, p.(Asp393Gly) *MTND5* mutation^15^. At the time of re-programming the patient fibroblasts exhibited heteroplasmy of 17% (patient A) and 55% (patient B). Multiple pluripotent clones were derived from the reprogramming and their heteroplasmy levels analysed. Heteroplasmy in each clone of the point mutation line (patient B) varied minimally (Fig. 1a), however the reprogramming of the deletion cell line (patient A) generated clones which all increased their heteroplasmy from 17% to circa 80% after which all clones died or spontaneously differentiated (data not shown). A second reprogramming of the deletion line A was performed and yielded three clones with heteroplasmies of <10% (A.1), ~30% (A.2) and ~65% (A.3) (Fig. 1a), this coincided with a switch to Nutristem media (Biological industries) which improved the survival of the clones, allowing them to stabilise and expand.

Marker analysis of clones from the two lines confirmed expression of markers consistent with all clones being pluripotent^16^ (Supplementary Fig. 1, Supplementary Table 1). Subsequent karyotyping of clone A.3 revealed a chromosome 20 re-arrangement, commonly associated with iPSC generation^17,18^, but this was not present in A.1 and A.2 or B.1. In a subsequent recloning of A.2 (see later) the same rearrangement was again observed only in clones carrying a significant load of mtDNA deletion (Supplementary Table 2). This alteration is likely to decrease the susceptibility to apoptosis in these cells^17,18^ and has not been linked to OXPHOS function.

### Clonal expansion in iPSCs with mtDNA deletions

Continuous sequential passage analysis of the A.1 (<10%) and A.2 (~30%) cells revealed a significant clonal expansion of the mtDNA deletion that was not observed in the A.3 (~65%) clone (Fig. 1a), in contrast to the point mutation clones which did not increase in heteroplasmy between passages regardless of their initial heteroplasmy (Green squares, Fig. 1a). Copy number analysis of the three deletion clones for the first five continuous passages only revealed a significant (p < 0.001) increase in total mtDNA copy number for clone A.3 (averaging 68% heteroplasmy), compared to clones A.1 or A.2 (Fig. 1b). Analysis of deletion to wild type mtDNA ratio for the 3 deletion clones reveals a proportionally greater increase in wild type mtDNA with increasing heteroplasmy (Fig. 1b), suggesting a compensatory mechanism to improve mitochondrial function in iPSCs carrying >60% heteroplasmy. In contrast, although the total copy number increased in clones from patient B, the number of wild type mtDNA molecules per cell was similar in the high and low heteroplasmy clones.

### Mitochondrial function is impaired in a heteroplasmy dependent manner

**Figure 2:**
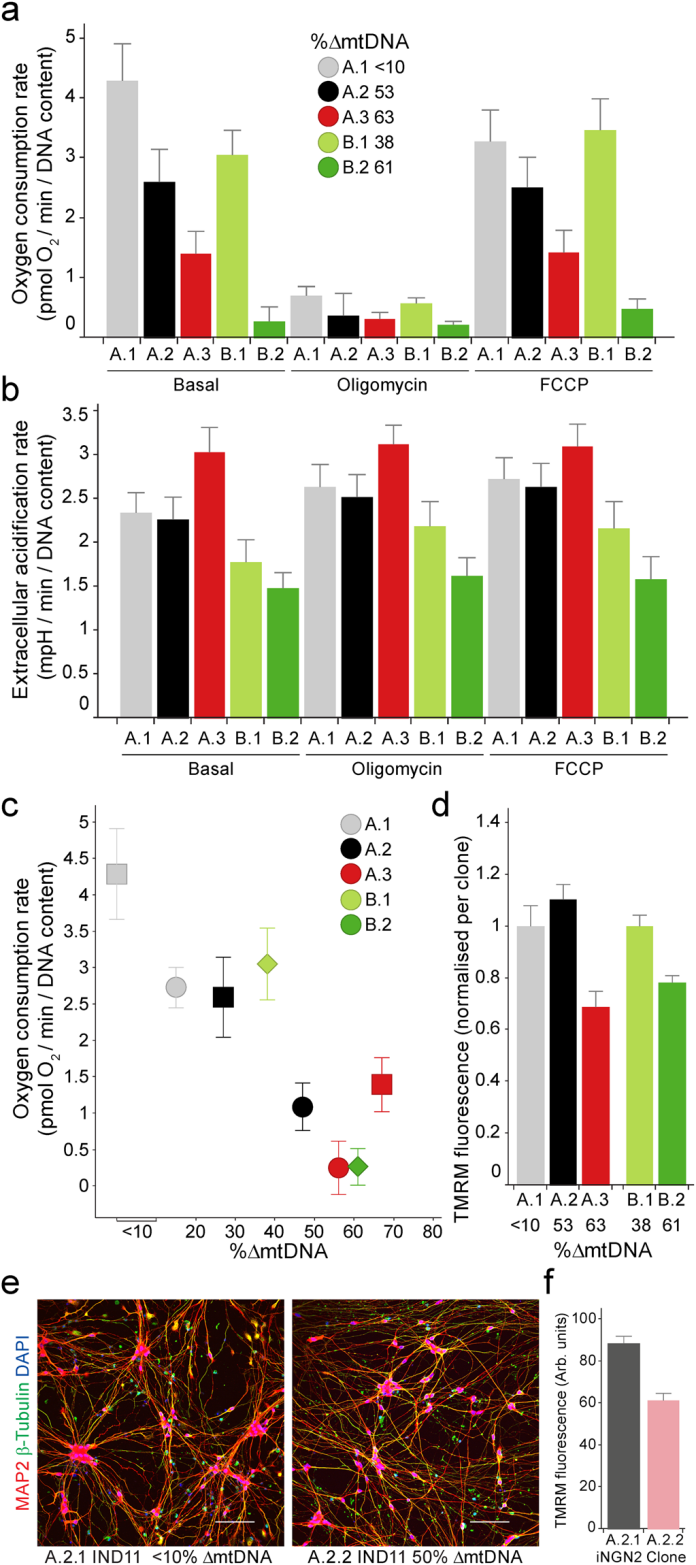
Heteroplasmy level correlates with mitochondrial phenotypes in iPSCs and iPSC-derived neurons. A.1 (grey), A.2 (black), A.3 (red), B.1 (light green) and B.2 dark green) iPSCs were assessed for (**a**) basal and maximal (FCCP) mitochondrial oxygen consumption (OCR; values corrected for non-mitochondrial OCR), and extracellular acidification (ECAR) as a proxy for glycolysis (**b**), and basal OCR compared to heteroplasmy (2 different passages per clone), (**c**) and (**d**) mitochondrial inner membrane potential (ψ) as visualized by TMRM staining. iNGN2 containing A.2 sub-clones A.2.1 and A.2.2 with <10% and 50% heteroplasmy respectively were differentiated for 11 (IND11) days and assessed for (**e**) neuronal markers using immunofluorescence (scale 10 µm) and (**f**) mitochondrial membrane potential using TMRM fluorescence. (**a–c**) mean values with s.d. n = 8 (biological replicates), (**d**) and (**f**) mean and s.e.m. **(d)** A.1 n = 10, A.2 n = 10, A.3 n = 10, B.1 n = 40, B.2 n = 40. (**f**) A.2.1 n = 93; A.2.2 n = 89.

To understand the effect of the mutations on mitochondrial function in iPSCs we determined mitochondrial oxygen consumption rate (OCR) and extracellular acidification rate (ECAR), a measurement of glycolytic respiration. Basal mitochondrial OCR decreased with increasing mtDNA heteroplasmy in both cell line A and B, as did maximal OCR after mitochondrial uncoupling by FCCP, indicating a decreased ability of cell lines harbouring high heteroplasmy levels to utilise oxidative respiration (Fig. 2a,c). Simultaneous measurement of ECAR highlighted an increase only in clone A.3, carrying 67% heteroplasmy, indicating that there is a requirement to upregulate glycolysis only when relatively high loads of heteroplasmy exist (Fig. 2b). Consistent with this observation, mitochondrial membrane potential was found to be lower in cells with high heteroplasmy (A.3 and B.2) when compared to their low heteroplasmy controls (A.1 and B.1) (Fig. 2d).

### Heteroplasmy stabilises in post mitotic neurons

iPSCs shift their metabolism from glycolysis towards oxidative respiration during differentiation, therefore we were interested to see if heteroplasmy increased as the iPSC differentiated into mature, post-mitotic neurons^19–21^. As the clones containing mtDNA deletions were the only cells to exhibit clonal expansion, we focused on these cell lines. We generated neurons by expressing inducible Neurogenin 2 (iNGN2) in the iPSC; this system was chosen for its ability to reproducibly produce high quality neuronal cultures^22^. Heteroplasmy analysis of 5 iNGN2 positive clones from the A.2 line (59% initial heteroplasmy) revealed a bimodal population, with low (n = 2; both <10%) and higher (n = 3; 55%, 68% and 70%) heteroplasmy. iNGN2 induction in one low (A.2.1 < 10%) and one higher heteroplasmy clone (A.2.2 55%) yielded cells with neuronal morphology that expressed β-tubulin 3 and Microtubule-associated protein 2 (MAP2) (Fig. 2e and Supplementary Fig. 2). Heteroplasmy levels were determined 8 and 30 days post-iNGN2 induction, with levels remaining constant in both the low and higher mtDNA heteroplasmy neurons (A.2.1 d8 =  <10%, d30 =  <10%; A.2.2 d8 = 50%, d30 = 48%) indicating no selection of mtDNA in either cell line post-differentiation. Consistent with the iPSC mitochondrial phenotype, the higher heteroplasmy clone A.2.2 (50%) displayed a lower membrane potential compared to the low heteroplasmy clone A.2.1 (<10%) (Fig. 2f).

### mtDNA deletions clonally expand *in vivo*

**Figure 3:**
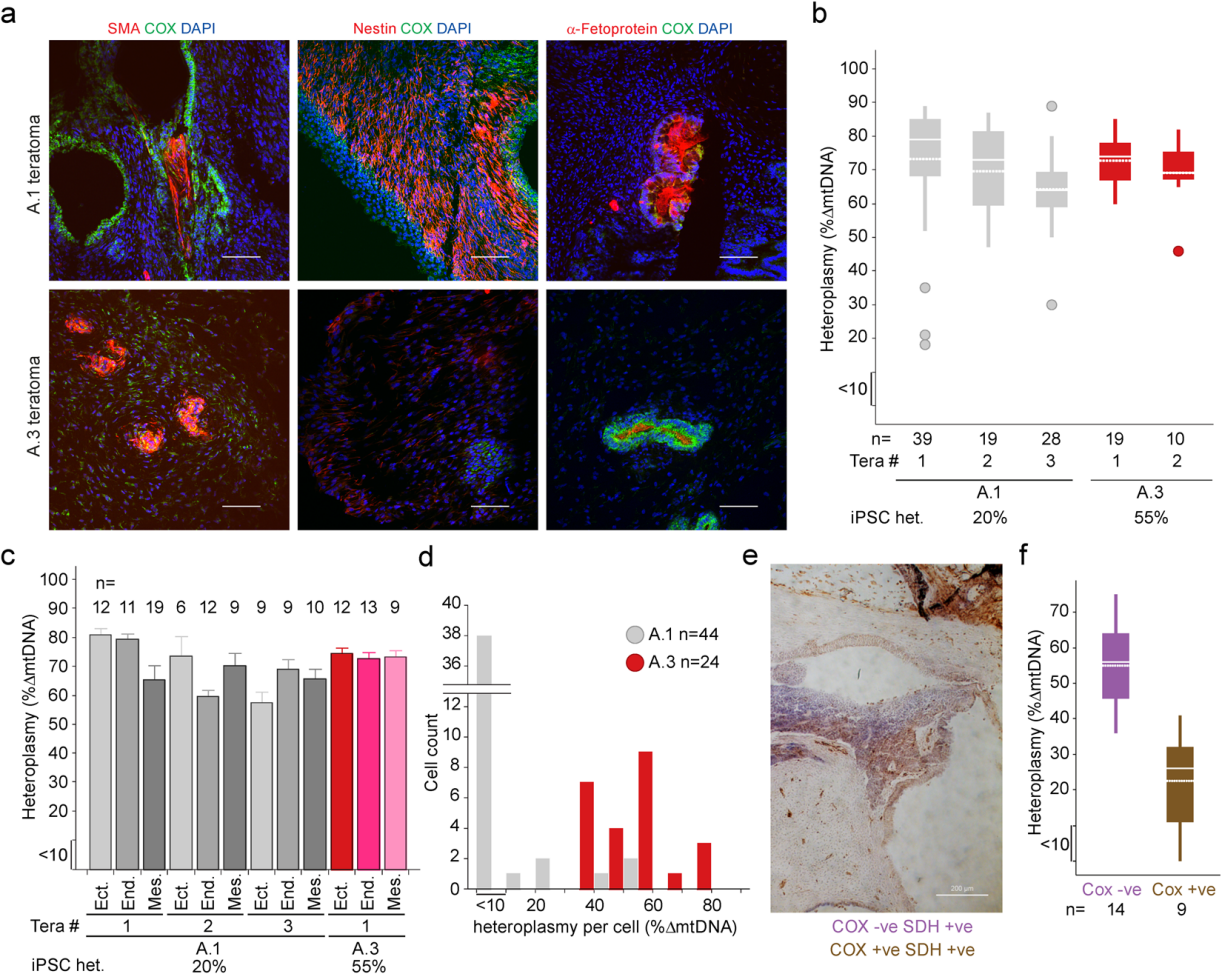
iPSCs carrying low or intermediate levels of heteroplasmy form teratomas in which all three germlayers carry high levels of heteroplasmy. NOD/SCID γ mice were inoculated with A.1 or A.3 iPSCs (20% and 55% heteroplasmy respectively) and the resulting teratomas were assessed for (**a**) germlayer markers smooth muscle actin (SMA) (mesoderm), Nestin (ectoderm) and α-fetoprotein (endoderm) (scale 10 µm). (**b**) Heteroplasmy was determined for 5 Teratomas – 3 formed using A.1 iPSCs (grey) and 2 formed from A.3 iPSCs. (**c**) Germlayers were laser microdissected to assess mean and s.e.m. for heteroplasmy from 4 fully differentiated teratomas – 3 formed from A.1 (grey shades) and 1 formed from A.3 iPSC (red shades). (**d**) Single cell heteroplasmy analysis of A.1 and A.3 iPSCs (heteroplasmy scores binned into 15 groups) (**e**) Following sequential COX/SDH histochemistry, regions of teratoma demonstrating COX-reactivity (brown) or COX deficiency (purple) were laser microdissected and (**f**) mtDNA heteroplasmy levels determined. (**b**) and (**f**) mean – dotted line; median – solid line; wide box - 1^st^ and 3^rd^ quartiles; circles – outlier values.

To assess mtDNA deletion clonal expansion *in vivo*, we created teratomas by injecting Non Obese Diabetic Severe Combined Immunodeficiency Gamma (NOD/SCID γ) mice (Jackson Labs) intra-testicularly with 2 × 10^5^ low (A.1 20%) or intermediate (A.3 55%) heteroplasmy iPSCs. To assess differentiation, embryonic germ layers were stained against nestin, α-fetoprotein and smooth muscle actin (SMA)^23,24^. An antibody selective of human COXIV was used to stain areas of non-murine origin (Fig. 3a and Supplementary Fig. 3). Three fully differentiated teratomas were formed by the A.1 line and one by the A.3 line. In a second A.3 teratoma not all three germ layers were detected due to the formation of a large cyst. Analysis of mtDNA deletion levels in multiple regions of these five teratomas revealed significantly increased heteroplasmy levels in all teratomas when compared to initiating iPSC mutant loads. Heteroplasmy of the three teratomas derived from the low heteroplasmy isogenic control A.1 cell line increased from an initial 20% to 73%, 70% and 64% respectively, teratomas formed by the intermediate A.3 line increased from 55% to 73% and 69% respectively (Fig. 3b).

In adult patients with single, large-scale mtDNA deletion disease high levels of mutation are detected in neuronal and muscular tissues^25^, therefore we analysed the heteroplasmy of the individual germlayers in each of the fully differentiated teratomas. Heteroplasmy increased above the starting iPSC levels in all germlayers to a similar high load (Fig. 3c). Interestingly no homoplasmic mutations were detected, reflecting the situation observed in human mtDNA deletion disease biopsies^13^ suggesting that there is a maximal level of heteroplasmy that these cells can tolerate *in situ*. The high heteroplasmy of the teratomas could be due to either clonal expansion of mutant mtDNA within single cells of the initiating pool of iPSC, or the teratomas being formed from a subset of initiating iPSC with high heteroplasmy. To distinguish these possibilities the single cell distribution of heteroplasmy in iPSCs A1 and A3 was assessed. A.1 cells predominantly carry very low levels of mutant mtDNA (86% < 10%), with no cell containing more than 50% heteroplasmy, whereas A.3 cells carry a range of heteroplasmy between 32% and 78% mutant mtDNA (Fig. 3d). As the teratomas all reached a similar size within a similar time period, the increase in heteroplasmy is likely due to mtDNA clonal expansion rather than selection of high heteroplasmy cells for teratoma formation.

### High heteroplasmy correlates with mitochondrial dysfunction in tissues

Sequential cytochrome *c* oxidase (COX) and succinate dehydrogenase (SDH) histochemistry was used to determine areas of mitochondrial dysfunction within the teratoma^26^ (Fig. 3e). Areas of positive COX reactivity (normal mitochondrial function) and COX deficiency in the presence of SDH reactivity (abnormal mitochondrial function) were observed throughout teratomas formed using both intermediate and low heteroplasmy iPSCs. Microdissection of these regions revealed that high mtDNA heteroplasmy segregated with areas of COX deficiency (Fig. 3f). Whilst the maximum mutation load detected in COX treated samples was lower than in immunofluorescent samples, this was likely due to low level contamination with mouse tissue, an unavoidable consequence arising from the absence of a specific marker of human tissue in this experiment.

### Clonal expansion occurs before differentiation

Cells retaining their pluripotency, as delineated by hNANOG staining^27^ in one teratoma derived from A.1 iPSCs, were found to have a heteroplasmy of 67% (±10%, data from 15 biological samples), suggesting that the accumulation of mutated mtDNA is occurring prior to differentiation into germlayers during stem cell replication and not clonally expanding in post-mitotic tissues. This is in agreement with our observation of stable heteroplasmy in cells differentiated into neurons in culture (see above).

## Discussion

Our findings show that iPSC lines with a single, large-scale mtDNA deletion undergo an increase in heteroplasmy with passage number. This increase occurs even though the deleted mtDNA has a phenotype in terms of a biochemical deficiency at high levels. The level of heteroplasmy did not change when the cells were differentiated into post-mitotic neurons after induction of NGN2 expression. However, when the iPS cell lines were injected into immunodeficient mice, the teratomas formed had high levels of deleted mtDNA (always greater than the starting level). This high level of heteroplasmy was also observed in undifferentiated cells within the teratoma suggesting that the clonal expansion has occurred prior to terminal differentiation of the initiating stem cells.

Whilst there have been few studies of single, large-scale mtDNA deletions in iPSCs, there have been a number of reports of different point mutations. Previous studies looking at iPSC with m.13513 G > A and m.8993 T > G mutations within protein encoding genes have also reported that high levels of heteroplasmy lead to a biochemical defect, however it was reported that heteroplasmy either remained stable or decreased overtime^2,5^. Several groups have generated iPSCs harbouring the m.3243 A > G tRNA mutation. A decrease in the heteroplasmy of m.3243 A > G iPSC cultured for one month has been reported by Perales-Clemente *et al*.^10^ whereas two groups^7,8^ have reported increasing heteroplasmy in some m.3243 A > G iPSC lines, however there was marked variability between clones^8^. As far as we are aware there has only been one report studying teratoma formation from m.3243 A > G cell lines, and the mutation load remained similar to that of the initiating iPSC in all six of the teratomas reported^4^.

*Cherry et al*. previously reported iPSC generation from a patient with a heteroplasmic 2.5 kb mtDNA deletion^6^. Two out of three cell lines generated in that study showed a consistent decrease in heteroplasmy over time, however one line was relatively stable and also showed a slight increase in heteroplasmy in later passages. Interestingly, the authors also reported difficulty in generating iPSC harbouring mtDNA deletions. This is similar to our experience and may be due to the greater pathogenicity of large scale mtDNA deletions. In our experience the change to Nutristem media improved the ability of iPSC to maintain pluripotency and heteroplasmy. Although it is not clear why this was the case, one explanation could be that Nutristem facilitates a metabolic shift towards glycolysis, lessening the impact of high mtDNA deletion heteroplasmy on the iPSC. Whilst these authors did generate teratomas from their deletion cell line they did not measure heteroplasmy so we do not know if the increase in heteroplasmy with the formation of teratomas is a consistent finding. The difference between our observations and those reported by Cherry *et al*. in cultured cells could be due to multiple factors including the different size of the deletion, the use of different culture media and the different nuclear background of the cells. Studies with other human mtDNA deletion iPSC lines would be helpful to study but this is challenging because mtDNA deletions tend to be lost in cultured somatic cells^6^.

Loss of large-scale mtDNA deletion is seen in patients with Pearson’s syndrome who survive. Pearson’s syndrome is associated with sideroblastic anaemia which improves with time and is associated with loss of mtDNA deletion in blood, but patients then develop Kearns-Sayre syndrome^28^. This loss of heteroplasmy in blood is also seen with other mtDNA mutations^29^ which suggests the cellular environment is very important as regards changes in heteroplasmy which makes our observations in teratomas particularly interesting.

There remains considerable debate about the mechanisms of clonal expansion with little evidence for the preferential replication of a smaller genome under normal circumstances^30,31^. However, studies by the Moraes group have shown that under circumstances where there is rapid mtDNA replication following mtDNA depletion, then the deleted form does replicate faster^32^. Therefore, one possibility is that under the highly glycolytic conditions found in culture and within the teratomas, a rapid replication of the mitochondrial genome is needed to keep pace with cell division and thus replication of the smaller genome is completed more often than the larger, wild type genomes. The fact that undifferentiated cells within the teratomas had high levels of heteroplasmy 67% (±10%, data from 15 biological samples), supports the idea that clonal expansion is occurring during stem cell division rather than in post-mitotic cells. The *in vitro* neuronal differentiation supports this conclusion as the over-expression of NGN2, a proneural protein active during embryonic development of neurons^33,34^, allowed the iPSC to rapidly differentiate from stem cells to post-mitotic neurons within 3 days (neurons were allowed to mature for a further 8 days, however the cells no longer divided after the 3 day NGN2 induction). In order to maintain mtDNA copy number during cell division, mtDNA is replicated in daughter cells prior to mitosis, therefore the more cell divisions that occur prior to terminal differentiation, the bigger the window available for an mtDNA species with a replicative advantage to clonally expand. The lower number of cell divisions (typically the iPSC will divide twice during the 3 day NGN2 induction) allowed us to initiate neuronal differentiation with low heteroplasmy levels. The longer half-life of mtDNA in terminally differentiated cells, which has been reported to be as long as 31 days^35^, could also explain the stability of heteroplasmy in the neurons generated from the iPSC as any kinetic selection of mtDNA would take much longer to become apparent.

The focus of our study was the effect of cell proliferation and differentiation on mtDNA deletion heteroplasmy. Work by other groups has shown that cellular metabolism shifts from glycolysis to OXPHOS during stem cell differentiation^36–38^ and although outside the scope of this work, the effect of the single large-scale mtDNA deletion on stem cell fate and lineage adoption could be investigated using this model by allowing iPSC with various levels of heteroplasmy, and thus varying degrees of OXPHOS deficiency, to differentiate in an uncontrolled manner followed by analysis of differentiated cell types.

One of the fascinations of mitochondrial DNA disease is the frequency of sporadic mtDNA deletions. Recent epidemiological studies confirm that single, large-scale mtDNA deletions affect about 17% of adults with mitochondrial disease and is far and away the commonest sporadic mtDNA mutation (most mtDNA point mutations are familial). This frequency of sporadic single, large-scale mtDNA deletions, compared to point mutations, must be due to either greater mutation rate and/or increased clonal expansion of deleted mtDNA compared to mtDNA containing point mutations. We know that mtDNA deletions must be present in oocytes from the finding of an identical deletion in monozygotic twins^39^, but observations show this can be at very low level^40^. One possibility is that the clonal expansion of the deletion occurs during the rapid cell replication phase in embryogenesis when the deleted mtDNA may have a kinetic advantage over its full-sized counterparts. Our data in teratomas would support this suggestion, and the generation of teratomas containing mtDNA deletions may prove to be a useful model to help our understanding clonal expansion in early embryogenesis. Whilst we appreciate that further work in this area is needed in order to understand the mechanism behind the preferential amplification of the deleted species, this work represents a valuable insight into the effect of rapid cell proliferation on mtDNA deletion heteroplasmy.

## Materials and Methods

### Cell lines

This study was approved and performed under the ethical guidelines issued by Newcastle University, the institution where samples were collected, (Newcastle and North Tyneside 1 and National Research Ethics Committee (reference 2002/205) and complied with the Declaration of Helsinki. Primary human dermal fibroblasts were obtained from three patients after obtaining appropriate informed consent. Patient A had Pearson’s syndrome and a ~6.0 kb single, large-scale mtDNA deletion (7777:13794) confirmed by Southern blotting to be a deletion rather than a duplication (Supplementary Fig. 4). Patient B had Complex-I deficient Leigh syndrome and a heteroplasmic m.13514 A > G, p.(Asp393Gly) MTND5 mutation. Cell lines were maintained in high glucose DMEM with GlutaMax (Gibco) supplemented with 1× sodium pyruvate (ThermoFisher cat# 11360-039) and 0.05 mg/ml uridine (Sigma# 3003). Cells were passaged prior to reaching confluence using TryplE express reagent (ThermoFisher Cat-# 12604013) for dissociation. All lines were maintained in a humidified atmosphere of 5% CO_2_ at 37 °C.

### Southern blotting

mtDNA was prepared from Patient A fibroblasts, a wild type fibroblast cell line and a muscle biopsy from patient M0488-14 carrying a known duplication and linearized with restriction enzymes *Pvu*II and *SnaB*I at 37 °C for 90 minutes. Samples were run on a 0.7% agarose gel and transferred onto a nylon membrane (Bio-Rad) overnight using SSC buffer. The radiolabelled probe was firstly PCR amplified from template DNA using the primers D1 R and F and 26 R and F and cleaned using ExoSAP (Promega)^41^. The probe was labelled using α-32dCTP and hybridised in the presence of salmon sperm DNA overnight at 65 °C in. The membrane was washed repeatedly in the presence of 1% SDS and SSC at 65 °C. The blot was developed in a Phosphoimager cassette overnight and visualised using the Typhoon FLA 9500 (GE Healthcare).

### Cell reprogramming, iPSC maintenance and neuronal differentiation

Primary human dermal fibroblasts from donors were used for reprogramming using Sendai virus with the help of the CytoTune-iPS reprogramming kit according to the standard protocol. Colonies with hallmark of pluripotent morphology were readily visible between days 17 and 20 after transduction. These were picked and sub-cloned multiple times on plates coated with Matrigel (BD Biosciences, San Jose, CA) in mTeSR medium until Sendai virus RNA could no longer be detected and the morphology looked stable. Pluripotency was controlled by FACS analyses (Supp. Fig. 1; Supp. Table 1). Karyotype analysis was performed by full-genome SNP analyses (Supp. Table 2).

### Maintenance of iPSC

iPSC were maintained on Matrigel coated dishes with NutriStem hESC XF medium (Biological Industries cat-# 05-100-1 A) supplemented with 1× sodium pyruvate and 0.05 mg/ml uridine. For passaging, cells were dissociated by TryplE express reagent and replated at a density of 1.5 × 10^4^ cells/cm^2^ in NutriStem with 10 µM Rock inhibitor (Y-27632) (Millipore# SCM075). Media was replaced with Rock inhibitor free NutriStem after 24 hours.

### Generation of iNgn2 iPSCs

Human Ngn2 cDNA was synthesized using sequence information from the Ensembl database (Ensembl Gene ID ENSG00000178403 or accession number NM_024019.3) and cloned under the control of TRE tight (Tetracycline Response Element) promoter in a PiggyBac/Tet-ON all–in-one vector. This vector contains a CAG rtTA16 cassette allowing constitutive expression of Tet-ON system and an Hsv-tkNeo cassette for generation of stable IPS clones^42^. For nucleofection; human iPSCs were adapted and maintained as feeder-free cultures on matrigel plate in NutriStem. After dissociation into single cells with TryplE express reagent approximately 1 × 10^6^ iPS cells were nucleofected by Amaxa nuclefector device using Human Stem Cell Nucleofector® Kit 1 (Lonza #VPH-5012) and Prg# B-016 with 4 μg of Ngn2 plasmid and 1 μg of the dual helper plasmid. Subsequently cells were replated on matrigel plates with NutriStem medium containing 10 μM of Rock inhibitor. Antibiotic selection (G418 0.1 mg/ml) was applied after 48 hours. Stable clones appear within 1 week.

### Differentiation of iNgn2 neurons

After dissociation, 1 × 10^6^ of iPS cells were plated on a 6 cm matrigel plate in proliferation medium (DMEM/F12 with Glutamax supplemented with 2% B27) (ThermoFisher, cat-# 17504-044) and 1% N2 (ThermoFisher, cat-# 17502-048), 10 ng/ml hEGF (ThermoFisher, cat-# PHG0315), 10 ng/ml hFGF (ThermoFisher cat-# CTP0263), 1% Pen/Strep (ThermoFisher cat-# 15070-063) and 0.05 mg/ml uridine containing Rock inhibitor (10 µM) for 1 d and doxycycline (1 µg/ml) for 3 d. Three days later, induced neurons were given differentiation medium (Neurobasal supplemented with 2% B27, 1% N2, Pen/Strep, 0.05 mg/ml uridine, 1 mM sodium pyruvate and the following growth factors at 10 ng/ml, BDNF (Cat.# 450-02), GDNF (Cat # 450-10), and hNT3 (Cat.# 450-03) (all from PeproTech).

### mtDNA heteroplasmy detection

DNA was extracted using a QIAamp DNA mini kit (Qiagen) for cell pellets (1 × 10^5^ cells) or by 20 μl single cell lysis buffer for single cell and teratoma analysis (50 mM Tris HCl (pH 8.5), 0.1% Tween 20, 0.2 mg/ml proteinase K (ThermoFisher, cat# 25530-049)) for 2 hrs at 56 °C followed by a 10 min, 95 °C denaturation step. Heteroplasmy of the point mutations was performed using a PyroMark Q24 pyrosequencer (Qiagen). All primers were designed to bind to mtDNA, accession sequence NC_012920.1. m.13514 A > G point mutants were amplified using primer pairs specific to the following regions; m.13455–13475, m.13539–13560 and pyrosquenced using primer m.13495–13513. m.11777 C > A point mutants were amplified using the primer pairs to m.11655–11674, m.11876–11900 and pyrosquenced using the primer m.11786–11802. Real-time PCR was used to determine the heteroplasmy of mtDNA deletion mutants using the Taqman assay previously described in^43^ on a StepOne Plus real-time PCR machine with the following modifications. Probes were designed with a non-fluorescent quencher and MGB moiety: MT-ND1 probe VIC-5′CCATCACCCTCTACATCACCGCCC-3′-MGB location m.3506–3529 and MT-ND4 probe FAM-5′-CCGACATCATTACCGGGTTTTCCTCTTG-3′-MGB location m.12111–12138. Standard curves were included for data analysis. mtDNA copy number was calculated by comparing the ratio of *MT-ND1* and the nuclear gene *B2M* (GenBank accession number: NG_012920) in a singleplex assay^44^. Primers and probe for the B2M assay were as follows: B2M forward primer specific to gene location n. 8969–8990, B2M reverse primer specific to n. 9064–9037 and B2M probe FAM-5′-ATGTGTCTGGGTTTCATCCATCCGACA-3′-MGB (n. 9006–9032). Final B2M primer and probe concentrations were 300 nM and 100 nM, respectively. The reaction volume was 20 µl and included 3 mM Mg^2+^. All real time PCR reactions were performed in triplicate. Below 10% heteroplasmy the assay becomes less accurate due to increasing measurement error. The reasons for this are unclear, therefore any sample with measured heteroplasmy of 0–10% was classed as <10%. The heteroplasmy is recorded if the standard deviation of the triplicate is <1Ct.

### Mitochondrial functional assays

For Seahorse experiments: Basal Assay medium and XF96 consumables were purchased from Seahorse Bioscience Inc. Oligomycin A, FCCP, rotenone and antimycin A were purchased from Sigma-Aldrich. Draq5 was purchased from Biostatus, Saphhyre700 from Li-Cor Bioscience. Oxygen consumption and glycolytic flux were measured with the Seahorse XF96 (Seahorse Bioscience) according to the manufacturer instructions. Briefly, cells were seeded at 450 × 10^3^ per well in Matrigel pre-coated Seahorse plate. 24 h post-seeding cells were then transferred in non-buffered Seahorse Assay medium containing 25 mM glucose, 1 mM sodium pyruvate and 2 mM of Glutamax. Coupled and uncoupled respiration was first measured by oligomycin A injection (1 µg/mL). Maximal respiration capacity was determined in 0.125 µM (5 µM for neurons) FCCP. Finally, non-mitochondrial respiration was determined using a combination of rotenone (1 µM) and antimycin A (1 µg/mL). Measurements were performed with cycles including 4 minutes of medium mixing followed by 3 minutes of measurements. For data normalization, cells were recovered after measurements by 4% final PFA fixation for 10 min. Cell nuclei were then stained for 1 h with a combination of 2 near infra-red dyes: Draq5 (1/10000) and Sapphyre 700 (1/1000) diluted in PBS +0.1% Triton. Stained cells were then detected with Odyssey Scanner (Li-Cor). Fluorescent intensity per well was used to normalize respiration values per well. The average of the non-mitochondrial respiration measures was then subtracted from each corresponding condition/timepoint per well; all similar condition measurements per well were then averaged. These means were compared across 8 biological replicates on a given day, to give the mean and standard deviation shown in the figures. ECAR values were similarly determined but the rotenone/antimycin values were not subtracted.

### Mitochondrial membrane potential

Cultured cells were incubated with 5 nM TMRM (Tetramethylrhodamine methyl ester; Thermo Fisher, cat# T-668) for 30 min at 37 °C. Imaging was carried out using an inverted Nikon Ti confocal microscope using 561 nm excitation. All images were captured using identical microscope settings. Four regions per well were captured and analysis of images was performed by drawing a region of interest around cells at random and measuring mean intensity using ImageJ software. Areas of the image that were absent of cells were used for background correction.

### Neuronal imaging

iNGN2 cells were seeded on glass coverslips in 6 well plates (5 × 10^5^ per well) after 3 days incubation with proliferation media. Cells were seeded into differentiation media and allowed to grow for 8 days, after which they were fixed in chilled 4% PFA for 30 min. Cells were permeabilised and blocked by 10% NGS (normal goat serum) (Sigma, cat# G9023) in 1% tween 20, TBS for 1 hr at room temperature. 1° antibodies (βtubulin 3 (Sigma, cat# T8578), MAP2 (Abcam, cat# ab32454) were diluted 1 in 500 in 3% NGS TTBS and incubated with cells overnight at 4 °C. Cells were washed 3× in TTBS and incubated with appropriate 2° antibodies (Thermo Fisher, cat# A21131, A11010) diluted 1 in 200 in 3% NGS TTBS for 90 min at room temperature, 10 µg/ml Hoechst was also present. Cells were washed 3× in TTBS and mounted on slides using ProLong Gold mountant (Thermo Fisher, cat# P10144). Images were captured using a Nikon Ti confocal microscope using 20× magnification.

### Teratoma formation

Teratomas were formed by inoculating NOD.Cg-Prkdc*scid* Il2rgtm1Wjl/SzJ (NSG) mice intratesticularly with 2 × 10^5^ iPSC cells suspended in a 1:1 mixture of mTesR1 and Matrigel. Animals were monitored daily and teratomas excised when they reached a sufficient size for analysis. All teratomas were excised by 10 weeks after injection. Upon excision, teratomas were halved and frozen in super cooled isopentane and stored at −80 °C prior to cryosectioning 15 µm sections on glass slides.

### Teratoma immunofluorescence and laser microdisection

Cut sections were fixed in chilled 4% PFA for 3 mins before permeablisation in a methanol series (70% MeOH 10 min, 95% MeOH 10 min, 100% MeOH 20 min, 95% MeOH 10 min, 70% MeOH 10 min). Sections were washed in TTBS and blocked in 10% NGS TTBS for 1 hour at room temperature (or 10% donkey serum (DS) for anti-nanog staining). 1° antibodies were against nestin (1:1000, Abcam, cat# ab6320), alpha fetoprotein (1:100, Abcam, cat# ab133617), smooth muscle actin (1:100, Abcam, cat# ab125044), nanog (1:40, R&D systems, cat# AF1997SP), COXIV + COXIVL2 (1:500, Abcam, cat# ab110261). Antibodies were diluted in 3% NGS TTBS and incubated overnight at 4 °C. Anti-nanog was diluted in 3% DS TTBS. Sections were washed 3× in TTBS and appropriate 2° applied in 3% NGS or DS TTBS with 10 µg/ml Hoechst for 90 min at room temperature. 2° antibodies used were all purchased from Thermo Fisher (cat#; A11056, A21123, A21131, A11010). After staining slides were washed 3× in TTBS and either mounted with ProLong Gold mountant on coverslips for imaging or washed 3× in ultrapure water and allowed to air dry in the dark prior to microdissection. Germlayers were laser microdissected using a Zeiss PALM microbeam. Dissected regions were lysed in 20 µl single cell lysis buffer and lysed as described above.

### SDH/COX staining

Glass mounted 15 µm cryosections were incubated in COX medium (100 μM cytochrome c, 4 mM diaminobenzidine tetrahydrochloride and 20 μg/ml catalase in 0.2 M phosphate buffer pH 7.0) at 37 °C for 50 minutes. Sections were washed 3× in phosphate buffered saline, pH 7.4 and incubated in SDH medium (130 mM sodium succinate, 200 μM phenazine methosulphate, 1 mM sodium azide, 1.5 mM nitroblue tetrazolium in 0.2 M phosphate buffer pH7.0) at 37 °C for 50 minutes after which sections were was 3× in PBS and either dehydrated in an ethanol series (70%, 95%, 2 × 100%), cleared in Histoclear (National Diagnostics) and mounted in DPX for imaging or dehydrated and allowed to air dry prior to laser microdisection as described above. Brightfield images of COX/SDH stained sections were captured using a Zeiss Imager M1, 20× magnification.

### Data Availability

No data sets were generated or analysed during the current study.

## Electronic supplementary material

Supplementary Information
