# Supplementary material for: Preferential amplification of a human mitochondrial DNA deletion in vitro and in vivo
Source: Sci Rep. 2018 Jan 29;8:1799. doi: 10.1038/s41598-018-20064-2 (PMC5789095; doi:10.1038/s41598-018-20064-2)
Supplement: Supplementary file 1 — Supplementary Information [file 41598_2018_20064_MOESM1_ESM.pdf]

Preferential amplification of a human mitochondrial DNA deletion *in vitro* and *in vivo*

Oliver M. Russell[1], Isabelle Fruh[2], Pavandeep K. Rai[1], David Marcellin[2], Thierry Doll[2], Amy Reeve[1], Mitchel Germain[2], Julie Bastien[2], Karolina A. Rygiel[1], Raffaele Cerino[2], Andreas W. Sailer[2], Majlinda Lako[3], Robert W. Taylor[1], Matthias Mueller[2], Robert N. Lightowlers[1], Douglass M. Turnbull[1,*], Stephen B. Helliwell[2,*]

Supplementary Information

Supplementary Figure 1

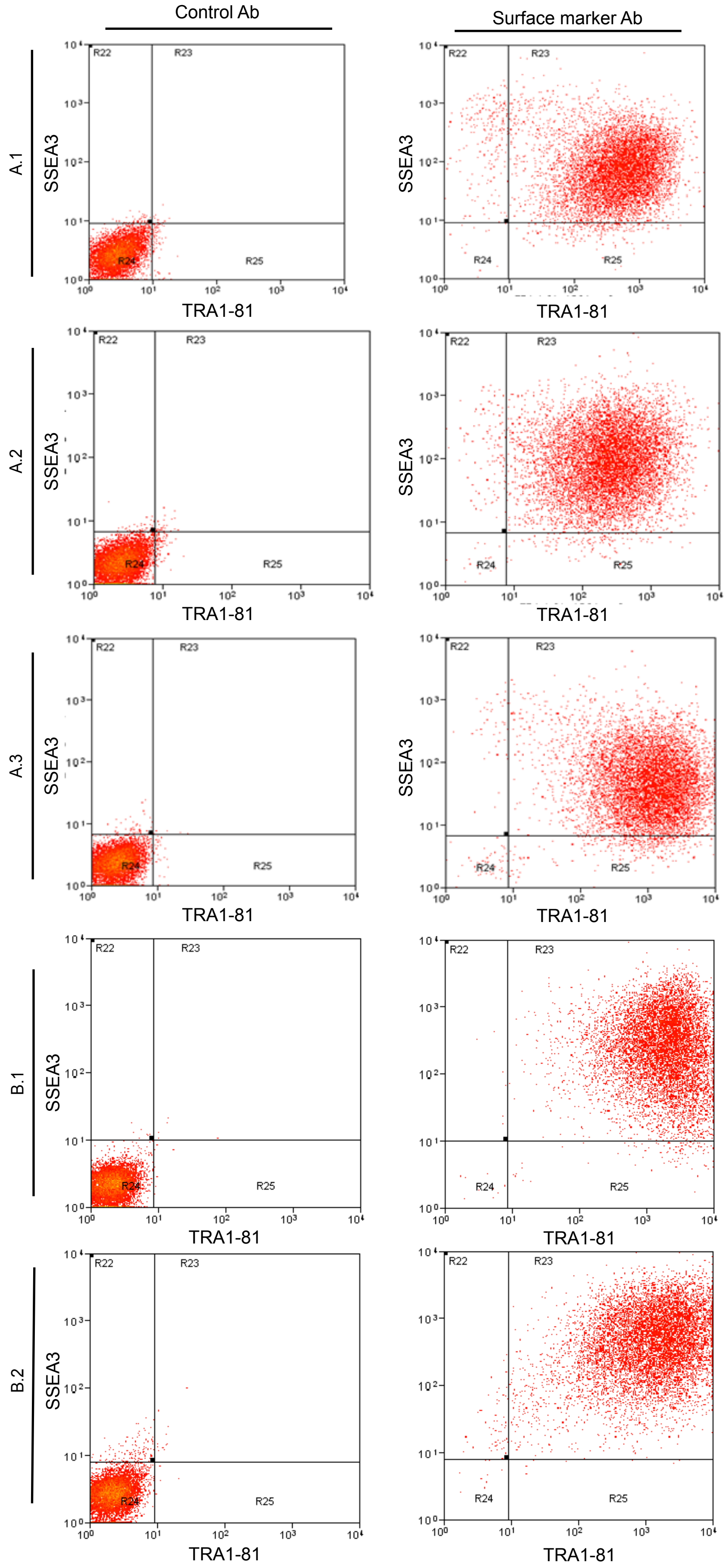

Supplementary Figure 1 iPSCs display typical pluripotency markers. FACS analysis using Abs for SSEA-3 and Tra1-81 with A.1, A.2, A.3, B.1 and B.2 iPSC clones, as compared to the same cells using isotype controls.

Supplementary Figure 2

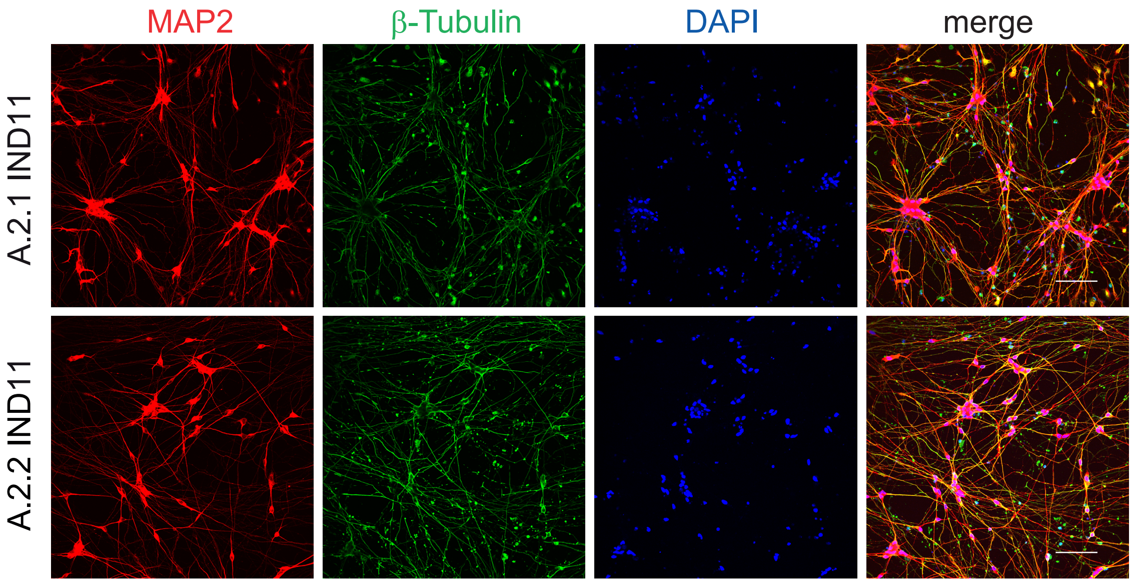

Supplementary Figure 2 iNGN2-induced neurons display typical neuronal markers. INGN2-containing A.2 sub-clones A.2.1 and A.2.2 with 0% and 50% heteroplasmy respectively were differentiated for 11 days and assessed for neuronal markers MAP2, alpha-Tubulin and DAPI using fluorescence microscopy. Merged image is identical to **Fig. 2e**

Supplementary Figure 3

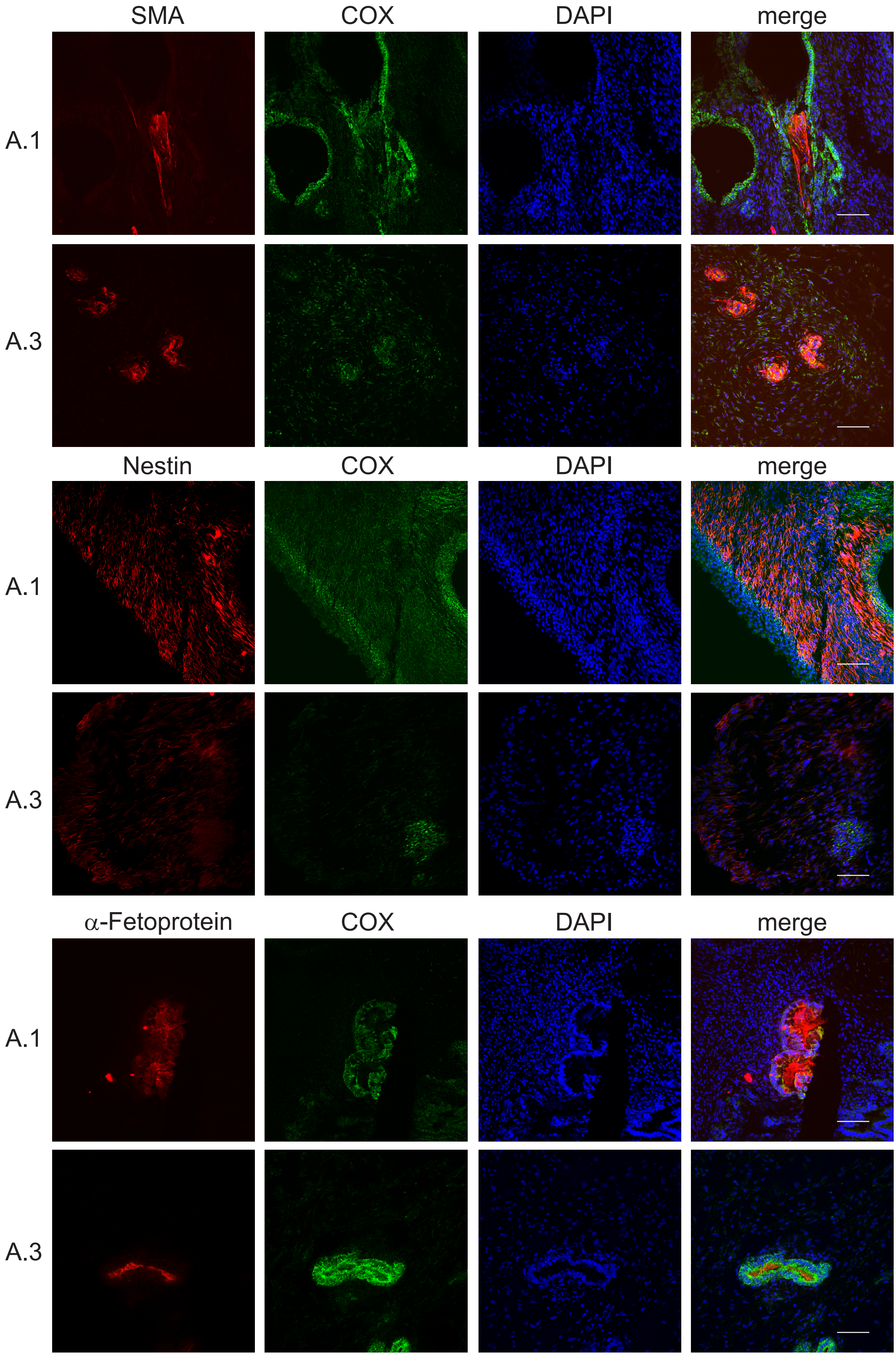

Supplementary Figure 3 iPSCs carrying low (A.1) or intermediate heteroplasmy (A.3) generate teratomas comprising all three germlayers. A.1 and A.3-derived teratomas were triple-stained for nuclei (DAPI), human mitochondria (COX) and either germlayer markers Nestin (ectoderm), alpha-fetoprotein (endoderm) or smooth muscle actin (SMA) (mesoderm); images shown for each colour channel and merged (merge). Merged panels are identical to those in **Fig. 3a**.

Supplementary figure 4

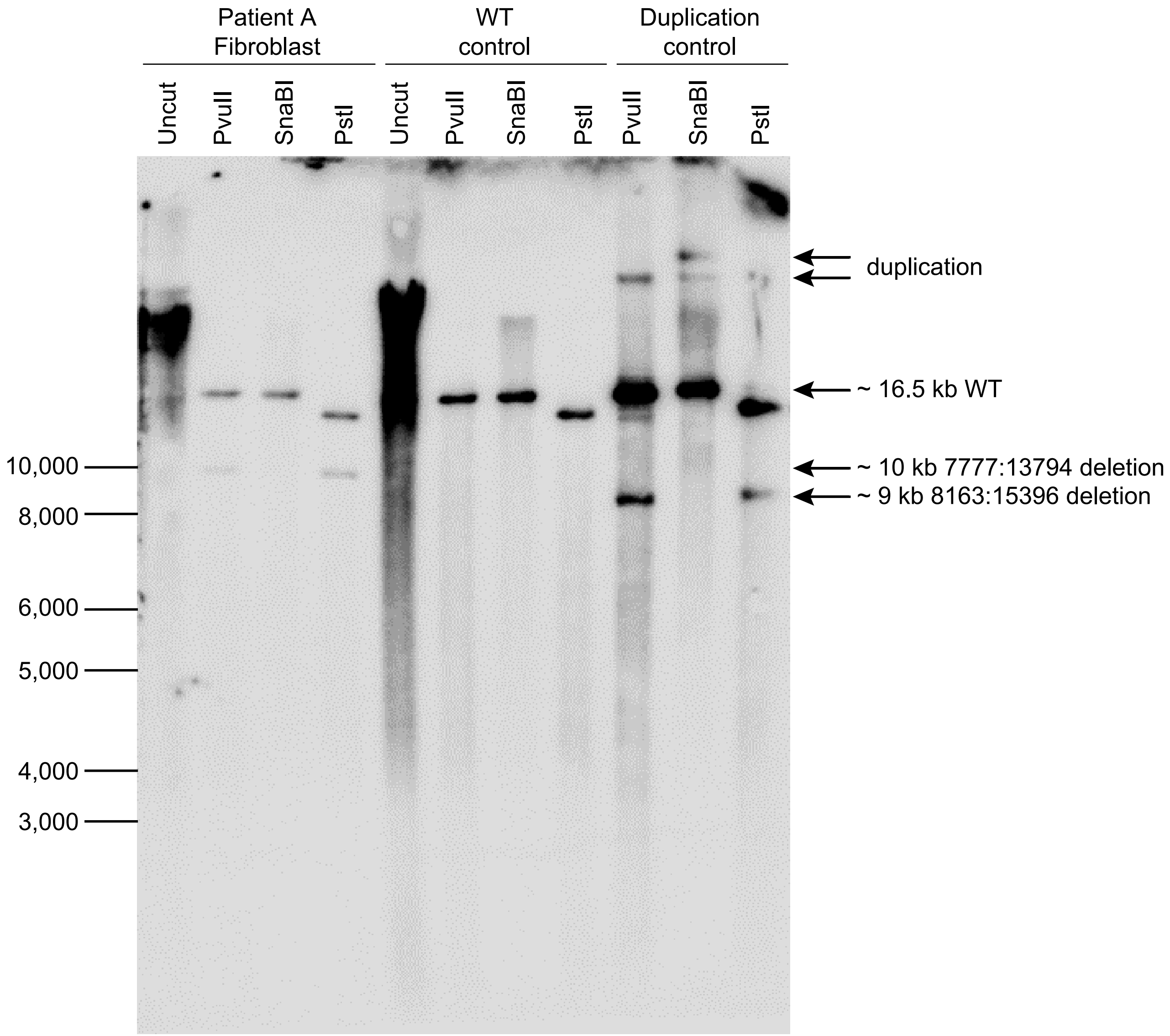

Patient A parent fibroblast: 6017 bp deletion; m.7777_13794 deleted, no obvious repeat breakpoints

WT control: Control fibroblast line

Muscle biopsy duplication control: 7,232 bp deletion; m.8163_15396 deleted, no obvious repeat breakpoints.

Supplementary Figure 4 mtDNA duplications are not detectable in Patient A fibroblasts. mtDNA from Patient A (7777:13794) parental fibroblasts, a control wild type fibroblast line, and a muscle biopsy sample carrying a duplication (8163:15396) was subjected to restriction digest analysis using enzymes noted in the figure. Predicted bands for the control and confirmed duplication cell lines are labelled. No duplication bands are detectable for Patient A fibroblasts.

Supplementary Table 1 | Summary of pluripotency and differentiation markers as assessed using FACS for A.1, A.2, A.3, B.1 and B.2

Line	Oct 4	Nanog	Sox2	SSEA-3	Tra1-80	SSEA-1
A.1	~90%	~90%	>90%	>90%	>90%	<20%
A.2	>90%	>90%	>90%	>90%	>90%	<20%
A.3	>90%	>90%	>90%	>90%	>90%	<20%
B.1	n.d.	n.d.	n.d.	>90%	>90%	<10%
B.2	n.d.	n.d.	n.d.	>90%	>90%	<20%

Supplementary table 2 | Karyotype of iPSC clones used in this study.

Name	Cell type	Gender	Report
	Fibroblast	female	No larger chromosomal aberrations observed.
A.1	iPS	female	Chr7 monosomy with loss of heterozygosity. Chr10: Onset of duplication mosaicism in telomeric region of short arm
A.2	iPS	female	Chr7 monosomy with loss of heterozygosity.
A.3	iPS	female	Chr20 single copy deletion of small arm, duplication of large arm.
A.2.1	iPS	female	Chr7 monosomy with loss of heterozygosity.
A.2.2	iPS	female	Chr7 monosomy with loss of heterozygosity. Chr20 single copy deletion of small arm, duplication of large arm.
B.1	iPS	Female	No larger chromosomal aberrations observed.
